# Carbohydrate-active enzymes from the zygomycete fungus *Rhizopus oryzae*: a highly specialized approach to carbohydrate degradation depicted at genome level

**DOI:** 10.1186/1471-2164-12-38

**Published:** 2011-01-17

**Authors:** Evy Battaglia, Isabelle Benoit, Joost van den Brink, Ad Wiebenga, Pedro M Coutinho, Bernard Henrissat, Ronald P de Vries

**Affiliations:** 1 Microbiology & Kluyver Centre for Genomics of Industrial Fermentation, Utrecht University, Padualaan 8, 3584 CH, Utrecht, The Netherlands; 2 Fungal Physiology, CBS-KNAW, Uppsalalaan 8, 3584 CT, Utrecht, The Netherlands; 3 Architecture et Fonction des Macromolécules Biologiques, UMR6098, CNRS and Universités d'Aix-Marseille I & II, Case 932, 163 Av de Luminy, 13288 Marseille cedex 9, France

## Abstract

**Background:**

*Rhizopus oryzae *is a zygomycete filamentous fungus, well-known as a saprobe ubiquitous in soil and as a pathogenic/spoilage fungus, causing Rhizopus rot and mucomycoses.

**Results:**

Carbohydrate Active enzyme (CAZy) annotation of the *R. oryzae *identified, in contrast to other filamentous fungi, a low number of glycoside hydrolases (GHs) and a high number of glycosyl transferases (GTs) and carbohydrate esterases (CEs). A detailed analysis of CAZy families, supported by growth data, demonstrates highly specialized plant and fungal cell wall degrading abilities distinct from ascomycetes and basidiomycetes. The specific genomic and growth features for degradation of easily digestible plant cell wall mono- and polysaccharides (starch, galactomannan, unbranched pectin, hexose sugars), chitin, chitosan, β-1,3-glucan and fungal cell wall fractions suggest specific adaptations of *R. oryzae *to its environment.

**Conclusions:**

CAZy analyses of the genome of the zygomycete fungus *R. oryzae *and comparison to ascomycetes and basidiomycete species revealed how evolution has shaped its genetic content with respect to carbohydrate degradation, after divergence from the Ascomycota and Basidiomycota.

## Background

The phylum Zygomycota is a primitive and early diverging group of fungi. Fungal species belonging to this basal fungal lineage are characterized by sexual reproduction via zygospores, asexual reproduction by uni-to multispored sporangia and, in most species, nonseptate (i.e coenocytic) hyphae. The Zygomycota is divided into two classes, the Zygomycetes and the Trichomycetes. The Zygomycetes are an ecologically diverse class of fungi, including both saprobes and pathogens of plants, animals (including humans) and other fungi. *Rhizopus oryzae *is a member of the order Mucorales, genus of *Rhizopus*. The order of Mucorales consists of genera such as *Mucor*, *Phycomyces*, *Rhizopus*, *Rhizomucor*. Although *Rhizopus *and *Mucor *are more closely related to each other than either of them to *Phycomyces*, they are not closely related genera [1]. *R. orzyae *has been reported as the predominant human-pathogen causing zygomycosis, a highly destructive and lethal infection on immune-compromised hosts [2]. In industry, it is one of the main *Rhizopus *spp. used in several traditional Asian fermented foods such as tempeh [3]. Furthermore, it is the well-known producer of metabolites such as organic acids (e.g. lactic acid, fumaric acid), ethanol and hydrolytic enzymes (e.g. glucoamylases, polygalacturonases) from pentose sugars and agricultural wastes such as barley, cassava, corn, potato pulp, oats and rice [4-9].

*R. oryzae *has a world-wide distribution with a high prevalence in tropical and subtropical regions. It has been isolated from many substrates, including a wide variety of soils, decaying vegetation, fruit, vegetables, seeds and dung [10]. As a fast growing filamentous fungus, it is known as a primary or secondary colonizer [11], invading quickly on easily accessible and digestible (e.g. rich in simple sugars) substrates. Recently the genome sequence of *R. oryzae *strain 99-880 has been published [12]. The genome paper focussed mainly on the evolution of the genome, which was shown to include a whole genome duplication event. Expansions of gene families have been observed for specific cellular processes related to the human pathogenic lifestyle such as cell growth and virulence. Little attention was given, however, to the ability of *R. oryzae *to degrade plant and fungal polysaccharides.

Several recent studies have demonstrated a strong relationship between the repertoire of carbohydrate active enzymes (CAZymes, http://www.cazy.org) [13] in fungal genomes and their saprophytic lifestyle [14-17]. These studies focussed in particular on those CAZymes involved in polysaccharide degradation. However, for *R. oryzae *only two CAZy enzyme families, GH18 (chitinases) [18] and GH28 (polygalacturonases) [19], have been studied in detail. Here we report the CAZy analysis of the *R. oryzae *and link this annotation to the ability of this fungus to use plant and fungal polysaccharides as carbon source. This will not only provide a better understanding of the ecological role of the *R. oryzae*, but also of sugar consumption and degradation by zygomycete fungi in comparison to ascomycete and basidiomycete fungi.

## Results and Discussion

### CAZy annotation reveals different numbers of CAZy families for *R. oryzae *in comparison to ascomycete and basidiomycete fungi

The putative CAZymes in *R. oryzae *99-880 were identified using the CAZy annotation pipeline (Additional File 1) and compared to a selection of ascomycete and basidiomycete fungi (Table 1).

**Table 1:** Comparative analysis of the number of putative genes in the 5 Carbohydrate-Active Enzyme http://www.cazy.org categories in 15 representative fungal genomes.

phylum	species	GH	GT	PL	CE	CBM	total	reference
*Zygomycota*	*Rhizopus oryzae*	116	130	6	41	24	**317**	[12]

	*Ustilago maydis*	102	64	1	18	12	**197**	[77]
	*Postia placenta*	248	102	8	25	34	**417**	[16]
*Basidiomycota*	*Phanerochaete chrysosporium*	181	66	4	17	48	**316**	[78]
	*Laccaria bicolor*	163	88	7	17	26	**301**	[17]
	*Schizophyllum commune*	237	75	16	30	30	**388**	[79]

	*Saccharomyces cerevisiae*	46	68	0	3	12	**129**	
	*Hypocrea jecorina*	193	93	6	17	48	**357**	
	*Nectria haematococca*	331	132	33	44	64	**604**	[80]
	*Gibberella zeae*	247	102	21	44	67	**481**	[81]
	*Podospora anserina*	226	86	7	41	97	**457**	[15]
*Ascomycota*	*Chaetomium globosum*	264	96	15	41	83	**499**	
	*Magnaporthe grisea*	232	92	5	47	65	**441**	[82]
	*Aspergillus fumigatus*	267	103	14	33	57	**474**	[83]
	*Aspergillus oryzae*	293	115	23	30	34	**495**	[84]
	*Aspergillus niger*	243	110	8	24	40	**425**	[85]
	*Penicilium chrysogenum*	216	101	9	22	49	**397**	[86]
	*Aspergillus nidulans*	251	91	21	31	41	**435**	[87]

GH: glycoside hydrolases, GT: glycosyl transferases, PL: polysaccharide lyases, CE: carbohydrate esterases, CBM: carbohydrate-binding modules in 15 fungal species. Numbers of *P. placenta *were from a dikaryotic strain (possibly 2 alleles per gene).

A total of 317 CAZymes have been identified in the *R. oryzae *genome. This value is similar to the number of CAZymes found in known plant cell wall (PCW) degrading basidiomycetes, higher than for yeasts, but significantly lower than for filamentous ascomycetes (Table 1). A total of 116 glycoside hydrolases were found in the *R. oryzae *genome, which is lower to what has been found in other filamentous fungi. In contrast, the total number of 130 glycosyl transferases is a very high value by comparison to other fungi (Table 1). Average numbers of polysaccharide lyases (PL), carbohydrate esterases (CE) and carbohydrate binding modules (CBM) were identified in *R. oryzae*.

In the next sections a more detailed comparison between the putative CAZymes in *R. oryzae *and ascomycete and basidiomycete genomes related to the degradation of PCW polysaccharides (cellulose, xyloglucan, β-1,3-1,4-glucan, xylan, galactomannan, starch, inulin and pectin) is described (Table 2). This analysis will be complemented by that of enzyme activities that may be involved in the metabolism of endogenous or exogenous fungal cell wall (FCW) polysaccharides. Although displayed for comparative purposes, the numbers of CAZy modules of the yeast *S. cerevisiae *are not included in the discussion, as this organism lacks most of the enzymes involved in the degradation of PCW polysaccharides. As a foreword, the tentative assignment of putative enzymes to specific metabolic pathways found below is meant to reveal the overall ability to deal individually with different polysaccharides. A more rigorous accounting, if ever possible, would only be possible following experimental characterization of many gene products. It should be noted that the CAZy annotations are based upon the protein models derived from the annotation of the genomes. As the pipelines from the JGI and the Broad Institute are not identical, this may cause small differences in the numbers per family that affected the comparison.

**Table 2:** Number of putative enzyme models for plant cell-wall degradation in different fungal genomes, assigned by substrate category.

species	cellu-lose	xylo-glucan	β-1,3-1,4-glucan	xylan	galacto-mannan	starch	inulin	pectin
*Rhizopus oryzae*	22	3	19	8	13	13	0	27

*Ustilago maydis*	19	3	38	15	15	10	2	15
*Postia placenta*	53	12	83	14	45	21	0	35
*Phanerochaete chrysosporium*	59	13	56	28	25	17	0	25
*Laccaria bicolor*	33	9	60	3	25	15	0	15
*Schizophyllum commune*	60	9	59	49	24	20	1	68

*Saccharomyces cerevisiae*	5	1	14	1	5	11	1	2
*Hypocrea jecorina*	33	11	35	26	28	11	0	21
*Nectria haematococca*	78	19	54	82	33	20	6	134
*Gibberella zeae*	59	16	48	56	30	19	5	87
*Podospora anserina*	72	8	39	55	26	17	0	47
*Chaetomium globosum*	84	12	42	65	24	17	2	66
*Magnaporthe grisea*	67	15	46	68	27	18	4	62
*Aspergillus fumigatus*	49	14	40	52	27	27	5	87
*Aspergillus oryzae*	51	17	38	63	27	30	4	109
*Aspergillus niger*	44	14	33	42	24	25	4	83
*Penicillium chrysogenum*	40	15	39	39	23	30	7	56
*Aspergillus nidulans*	55	16	38	52	36	25	2	94

The number of enzymes in the different functional categories is only a rough estimate and not an absolute count, as functional assignments are sometimes based on remote sequence similarities to biochemically characterized enzymes. Total values obtained per substrate correspond to the theoretical maximum number of active enzymes that could be obtained by adding up all the members of the concerned families (as described in Suplementary Table 2). These values are to be taken as a maximum rather than as absolute values. For Postia placenta, absolute values from the genomic effort on a dikaryon are provided but a monokaryon is estimated to correspond to approximately to 70% of the total provided (Coutinho and Henrissat, unpublished).

### CAZy annotation of the *R. oryzae *genome indicates highly specialized plant polysaccharide utilisation

#### Cellulose degradation

Degradation of cellulose is achieved by the synergistic action of endoglucanases (endo-β-D-1,4-glucanases) and cellobiohydrolases, assisted by β-glucosidases. It should be noted that β-glucosidases also have other functions than to assist cellulose degradation and therefore not all β-glucosidases may be involved in cellulose breakdown. A total of 21 and 19 candidate cellulolytic enzymes are found in *R. oryzae *and in *U. maydis *genomes, respectively, lower than what is observed for the other species (Table 2). *P. anserina *had the largest set of candidate enzymes (72) related to cellulose degradation, as shown previously [15].

Fungal cellobiohydrolases are classified in families GH6 and GH7 and β-glucosidases are found in families GH1 and GH3. The *R. oryzae *genome contains no protein models belonging to families GH1, GH6, GH7. Moreover, only six GH3 genes were identified in *R. oryzae *compared while filamentous ascomycetes typically contain more than ten (Table 2). None of the GH3 members were predicted to be β-glucosidases (Additional File 1).

*R. oryzae *contains the largest number of putative enzymes assigned to family GH45 (5) whose characterised members are typically endoglucanases (Additional Files 1 and 2). Three out of these five GH45 ORFs contain a N-terminal CBM1 module (Additional File 1) and have previously been shown to encode functional endoglucanases (*rce1*, *rce2 *and *rce3*) [20,21]. GH45 endoglucanases with a similar N-terminal CBM1 module have been identified in other members of the Mucorales [22], including MCE1 and MCE2 from *Mucor circinelloides *[23] and PCE1 from *Phycomyces nitens *[24]. Interestingly, the *R. oryzae *genome contains four putative protein models of GH9 (Supp. Table 2), a family of endoglucanases mostly found in bacteria, plants, and occasionally in animals [25]. GH9 members are absent in all ascomycete filamentous fungi, while basidiomycetes generally encode a single GH9 protein harbouring a C-terminal membrane spanning region (Additional File 2). The role of fungal GH9s remains unclear, but is probably unrelated to cellulose degradation [26]. Finally, *R. oryzae *does not appear to encode any family GH61 protein, which have been shown to boost cellulose breakdown by cellulases [27,28].

Altogether the analysis suggests that the main cellulose-degrading enzymes produced by *R. oryzae *are GH45 endoglucanases and that the fungus uses them solely to access the plant material. Cellulose is therefore probably not a nutritional source for *R. oryzae*, as enzymes able to fully degrade cellulose into glucose have not been found.

#### Xyloglucan

The *R. oryzae *genome is poor in protein models related to xyloglucan degradation (3) (Table 2). Only *U. maydis *had a similarly low number, while the other basidiomycete and ascomycete fungi contain 8 to 17 protein models (Additional File 2). The genome appears to contain no xyloglucan-active β-1,4-D-endoglucanase (GH12, 74) nor any side-chain cleaving enzymes such as α-L-fucosidases of families GH29 and GH95 (Additional File 2). The 3 members of family GH31 are candidate α-glucosidases and not likely α-D-xylosidases (Additional File 1).

#### β-1,3-1,4-glucan degradation

The *R. oryzae *genome also encodes only a relatively small number of enzymes for the breakdown of β-1,3-1,4-glucans (Table 2). 1,3-1,4-β-Glucans, which are abundant components of the cell walls of grasses [29], are typically hydrolysed by lichenases and endo-1,3-1,4-β-glucanases of family GH16. Eight GH16 protein models were identified in *R. oryzae*, a low number compared to ascomycete and basidiomycete species (Table 2).

#### Xylan degradation

**Figure 1:**
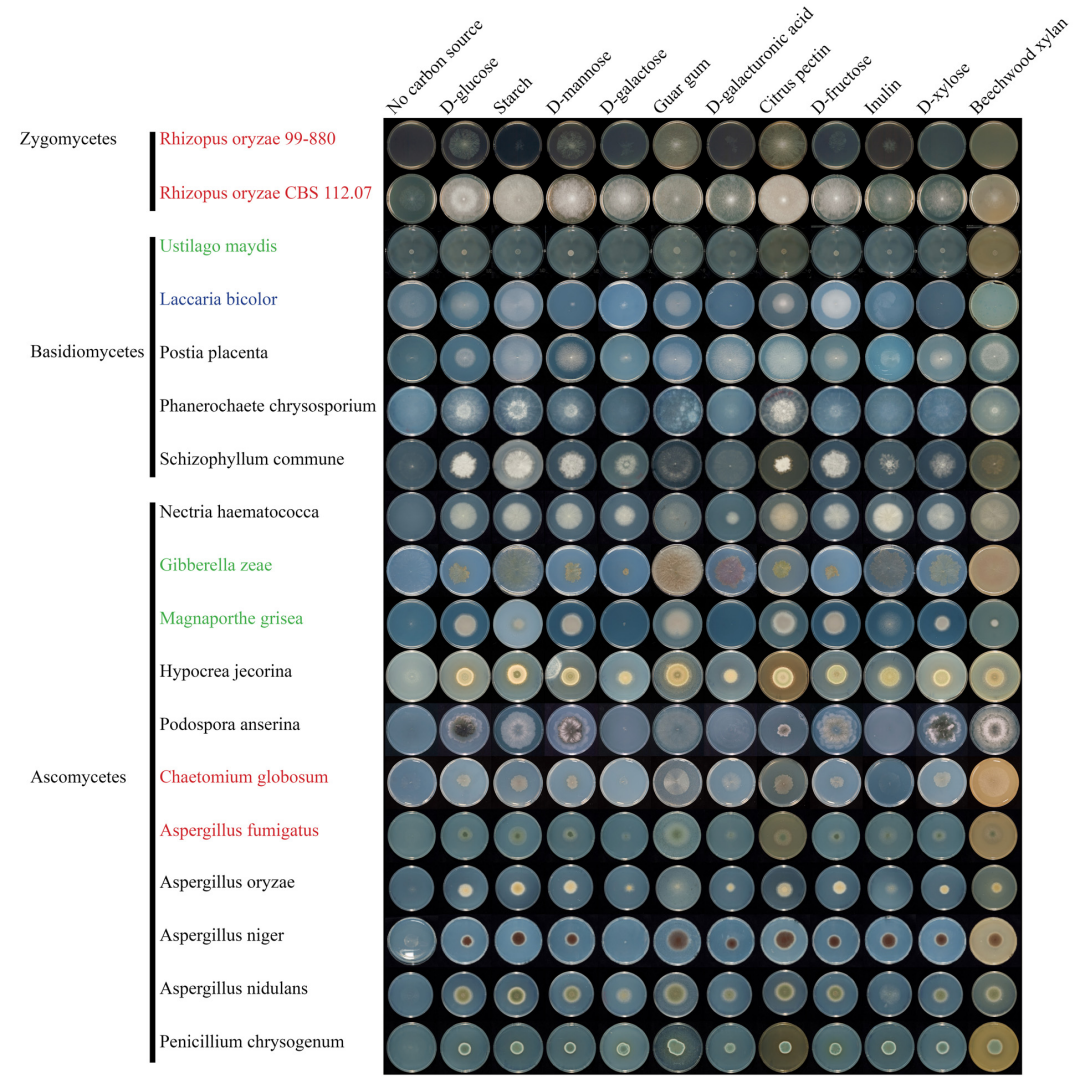
Growth of *R. oryzae *and 17 basidiomycete and ascomycete species on monosaccharides (D-glucose, D-mannose, D-galactose, D-galacturonic acid, D-fructose and D-xylose) and plant polysaccharides (starch, guar gum, citrus pectin, inulin and xylan).

Endoxylanases, commonly found in families GH10 and GH11, cleave the xylan backbone into smaller oligosaccharides, which are degraded further by β-xylosidases to xylose [30]. The *R. oryzae *genome contains no putative ORFs belonging to either family GH10 or GH11. However, it has been demonstrated that *R. oryzae *CBS 112.07 produces a low molecular weight endoxylanase [31]. Interestingly, family GH3 or GH43 candidate β-xylosidases were detected in the genome (Additional File 2). The two GH43 members were functionally annotated as candidates for arabinan endo-1, 5-α-L-arabinosidases (Additional File 1). *R. oryzae *appears to be unable to breakdown xylan significantly as it lacks essential xylanolytic enzymes.. With the exception of *R. oryzae *and the mycorrhizal basidiomycete *L. bicolor *(Figure 1, Additional File 2 and [17]), all other analyzed fungal species contain putative endoxylanases from families GH10 and GH11. The latter two fungi appear to share the inability to degrade xylan with the oomycete *P. ultimum *[17,32].

#### Galactomannan degradation

The *R. oryzae *genome contains 13 candidate CAZymes related to the degradation of the main chain and side-chains of galactomannan. This value is similar to that of *U. maydis*, but significantly lower than that found in the other analyzed species (Table 2). No clear candidate β-endomannanase (EC 3.2.1.78) or β-mannosidase (EC 3.2.1.25) from families GH2, GH5 and GH26 were found in *R. oryzae. *Among the few GH5s found in *R. oryzae*, only one was found to be distantly related to bacterial β-mannosidases (Additional File 1). This constitutes presently the unique candidate enzyme for a possible role in the breakdown of the (gluco)mannan backbone. In *A. niger*, the presence of a single characterized β-mannosidase (MndA) and β-mannanase (Man5A) has been shown to be sufficient for an efficient degradation of mannan from different sources [33,34].

The genome *R. oryzae *contains a total of six protein models in families GH27 and GH36, mostly described as α-galactosidases (Additional File 1). The enrichment of α-galactosidases in the genome suggests galactose release by *R. oryzae*, probably for nutritional use, from structural or storage galactoglucomannan, oligosaccharides such as melibiose, raffinose, stachyose, and/or xylan or xyloglucan.

#### Starch and inulin degradation

The *R. oryzae *genome contains an average number of candidate CAZymes involved in starch degradation compared to the ascomycetes and basidiomycetes (Table 2). *R. oryzae *contains the highest number of GH15 enzymes (6), whereas few members from families GH13 (4) and GH31 (3) were detected (Table 2). Four of the six GH15 members were annotated as candidate glucoamylases or related enzymes (Additional File 1). Glucoamylase (AmyA; RO3G_00082.3) has been characterized previously [35], in part due its common usage in the starch industry because of its high activity and stability [36]. One of them has been previously characterized as a glucoamylase (AmyA; RO3G_00082.3) [35] and this enzyme has been commonly used in industry because of high activity and stability [36]. AmyA consists of a C-terminal catalytic domain, connected to an N-terminal starch-binding module of CBM21 by an O-glycosylated linker [37]. Interestingly, a very high number of CBM21-containing protein models (8) containing a CBM21 module were observed in *R. oryzae *compared to ascomycetes and basidiomycetes (1 - 4) (Additional File 1). Except for AmyA the actual function of these putative proteins is unknown. No catalytic modules were detected for the other seven ORFs, but all eight ORFs contain a putative phosphatase regulatory subunit.

Candidate starch-binding CBM20-containing protein models are found in all ascomycetes and basidiomycetes, excepting *U. maydis*. No CBM20 module is present in the *R. oryzae *genome, a feature probably functionally compensated by the eight CBM21-containing proteins. Interestingly, most CBM21-containing proteins are found among Eukaryota, contrasts with CBM20s that are equally dispersed among Bacteria and Eukaryota. As expected, none of the other CBM families containing starch-binding modules (CBM25, CBM26, CBM34 and CBM41) were found in the species analysed in this study. They are only found in prokaryotes (mostly Eubacteria) and the only one in Eukaryotes is in picophytotoplanktonic green algae [38].

No clear candidate invertases or inulinases could be detected in the genome of *R. oryzae*. In the *R. orzyae *NBRC 4785 strain, a glucoamylase (similar to *R. oryzae *NRRL 395 *amyB *[39]) with sucrose-hydrolyzing activity has been however reported [40]. AmyB has no starch-binding module and no apparent glucoamylase activity, but it was highly similar to the catalytic module of AmyA (glucoamylase). Only the equivalent AmyA mentioned earlier is found in the genome of *R. oryzae *strain 99-88 suggesting strain variations within family GH15. Interestingly, *Aspergillus *starch-degrading glucoamylases have been known for their promiscuity in degrading substrates containing α-linked D-glucosides [41] but no other reports of an ability to degrade sucrose are known.

#### Pectin degradation

In the *R. oryzae *genome, pectin degradation appears to be the focus of the highest number of putative CAZymes (27 predicted enzymes in Table 2). In comparison to the ascomycete and basidiomycete species, this is an average value. Less pectin-degrading CAZymes were predicted for *U. maydis *and *L. bicolor*, while the highest potential for pectin degradation is found in *A. nidulans *and *A. oryzae *(with an estimation of 94 and 109 CAZymes each, respectively). The *R. oryzae *genome is especially rich in family GH28 protein models, with twelve and three genes encoding candidate endopolygalacturonases (EC 3.2.1.15) and exopolygalacturonases (EC 3.2.1.67), respectively [19]. Endopolygalacturonases particularly hydrolyse the α-1,4 linked D-galacturonic acid within the homogalacturonan chain and exo-polygalacturonases hydrolyse this chain from the nonreducing end [30]. Our analysis shows that the enrichment of main-chain degrading enzymes of pectin was accompanied by low numbers of accessory enzymes (Table 2). A possible explanation is that *R. oryzae *is limited in the degradation of unbranched pectins, unlike most ascomycete and basidiomycete fungi that can degrade a variety of lowly and highly branched pectins.

This view fits well with the pre- and/or post harvest pathogenic lifestyle of *R. oryzae*. As a causal agent of Rhizopus rot, *R. oryzae *produces endopolygalacturonases for maceration of plant seedlings, fruits and vegetables and plant structures such as flowers, bulbs and tubers [42-44]. The pectin structure in berries, black currants and apples has been shown to contain a high amount of D-galacturonic acid and a small amount of rhamnose residues, indicating the presence of unbranched pectin [45,46]. Rhizopus rot is characterized by the dissolution of the PCW middle lamella in fruits and vegetables, that are typically rich in unbranched pectin (homogalacturonans) with a low degree of methyl esterification [47]. Such dissolution would facilitate accessibility into plant tissue. The natural ability of *R. oryzae *to degrade unbranched pectin has been exploited in industry, where GH28 endopolygalacturonases has been shown to be very useful in flax-retting (i.e. a process that degrades the smooth regions of pectin for separation of plant fibres) [48-50].

In summary, the genome contains mainly enzymes involved in degradation of storage polysaccharides (galactomannan and starch) and the backbone PCW structural polysaccharides (cellulose and pectin). The apparent absence of (i) exo-acting enzymes for cellulose, (ii) accessory enzymes for pectin degradation, and (iii) any enzymes for degradation of xyloglucan, β-1,3-1,4-glucan, xylan and inulin, suggest that these polysaccharides do not serve *R. oryzae *as a major carbon source. The degrading capacity of *R. oryzae *allows rapid invasion on and into the substrate, outcompeting secondary and late colonizers that will have to cope with a more extensive degradation of more resistant complex PCW polysaccharides.

### The hydrolytic potential of the *R. oryzae *genome correlates with growth on polysaccharides and related monosaccharides

The CAZy annotation results were compared to the growth profile of two *R. oryzae *strains (the sequenced isolate 99-880 and the type strain of the species CBS 112.07) and a selection of species from the Fung-Growth database http://www.fung-growth.org on a number of carbon sources (Figure 1). Overall growth of the type isolate was better than the sequenced isolate. A strong preference for citrus pectin and guar gum (a galactomannan) was observed for *R. oryzae *99-880, followed by inulin and starch. This pattern was largely the same for the type isolate, but this strain grew better on starch than the sequenced strain. Good growth on pectin, galactomannan and starch correlates with the presence of related CAZymes in the genome and the occurrence of *R. oryzae *on fruits, vegetables and seeds [10]. Fruits and vegetables are rich in pectin, whereas seeds are enriched in storage polysaccharides such as galactomannan, starch and inulin. Both *R. orzyae *strains were able to grow on inulin, despite the absence of known genes required for its degradation. Genome annotation of the oomycete *Pythium ultimum *initially also identified no candidate invertase [32]. An invertase gene was detected after Blast analysis of the raw nucleotide sequence, indicating that this was missed by the model-building pipeline. In light of this we used the same approach, but failed to identify any putative invertase in the sequenced strain of *R. oryzae*. Alternative explanations for the growth on inulin could be the presence of traces of other carbon sources in the commercial inulin preparation, or the presence of yet to discover fructofuranosidases.

No to poor growth (similar to growth on agar without carbon source) was observed on xylan (Figure 1) and cellulose (data not shown). Xylan was utilized by all other tested species with the exception of *L. bicolor *(Figure 1). This is in agreement with the absence of genes required to degradation of xylan in the genomes of *R. oryzae *and *L. bicolor*, and presence in all other tested genomes.

Growth on citrus pectin, guar gum, starch and inulin correlated well with growth on their associated monosaccharide components. *R. oryzae *showed average to good growth on D-galactose and D-mannose, the main components of galactomannan (Figure 1). The pectin main chain is mainly composed of D-galacturonic acid residues with small amounts of L-rhamnose residues, while the side chains consist of L-arabinose and D-galactose [30]. This correlates well with the ability of *R. oryzae *to grow on D-galacturonic acid (Figure 1) and L-rhamnose (data not shown). No growth of *P. chrysogenum*, *L. bicolor, P. anserina *and *M. grisea *was observed on D-galacturonic acid (Figure 1). In general, fungi do not prefer D-galacturonic acid as a carbon source (de Vries et al, unpublished results). No to poor growth occurred on L-arabinose or D-ribose (data not shown), which strongly suggests that pentose sugars are not preferred by *R. oryzae*. Growth on D-xylose was observed for *R. oryzae *CBS 112.07, which confirms a previous study [51]. However, no growth was observed for 99-880 on this substrate, demonstrating a significant difference between the isolates for xylose utilisation. Part of this difference may be due to a poor ability of some *R. oryzae *strains to germinate on D-xylose [8].

### CAZy annotation reveals specific differences in the chitinolytic and glucanolytic enzyme system for *R. oryzae*

The following sections describe the comparison of the number of CAZy family members between *R. oryzae *and the ascomycete and basidiomycete species that are implicated in the turnover/modification of the fungal cell wall (FCW) polysaccharides chitin, chitosan, α-1,3-glucan, β-1,3-glucan and β-1,6-glucan (Table 3). *R. oryzae *appears to contain over 40 CAZymes for chitosan degradation/biosynthesis, more than any of the species analyzed so far. The chitin recycling machinery found in *R. oryzae*, is similar in size to that observed in filamentous ascomycete and basidiomycete species (Table 3). Significantly, low numbers of CAZymes for the degradation of α-1,3-glucan, β-1,3-glucan and β-1,6-glucan were found in *R. oryzae *when compared to ascomycetes and basidiomycetes.

**Table 3:** Number of putative enzyme models related to fungal cell wall modification and recycling in different fungal genomes.

species	chitin	chitosan	α-1,3-glucan	β-1,3-glucan	β-1,6-glucan
*Rhizopus oryzae*	21	43	5	27	7

*Ustilago maydis*	8	20	4	43	14
*Postia placenta*	31	43	15	98	42
*Phanerochaete chrysosporium*	19	34	10	59	21
*Laccaria bicolor*	16	33	10	69	30
*Schizophyllum commune*	20	29	15	72	21

*Saccharomyces cerevisiae*	2	7	2	21	5
*Hypocrea jecorina*	26	20	9	60	13
*Nectria haematococca*	34	32	15	94	18
*Gibberella zeae*	24	27	9	73	15
*Podospora anserina*	22	27	9	57	15
*Chaetomium globosum*	19	27	10	59	12
*Magnaporthe grisea*	20	30	9	72	14
*Aspergillus fumigatus*	25	28	15	65	14
*Aspergillus oryzae*	22	24	19	66	13

See note in Table 2 on the confidence of the assignments to functional categories. Numbers of *P. placenta *were from a dikaryotic strain (possibly 2 alleles per gene).

#### Chitosan and chitin degradation

Chitin deacetylases catalyze the deacetylation of chitin to chitosan. In *R. oryzae*, a very large set of chitin deacetylases of family CE4 is found (Additional File 2). With a total of 34 CE4 models, this is more than three times the previous highest number of CE4s observed in the basidiomycete *L. bicolor *or in the ascomycete *M. grisea*. Most of the CE4 proteins in *R. oryzae *appear to be GPI-anchored (Additional File 1). The biosynthesis of cell wall chitosan, the main cell wall component of zygomycete species [52,53], proceeds by coordinated action of both chitin synthase and chitin deacetylases [54]. The many chitin deacetylases in the *R. oryzae *genome complement a large set of chitin synthases, previously reported as an expansion compared to ascomycete genomes [12]. The physiological role of chitin deacetylases in *R. oryzae *is presently unknown but they have been suggested to play roles in FCW biosynthesis in *Mucor rouxii *and *Absidia coerulea *and in plant-pathogen interactions in *Colletotrichum lindemuthianum *and *Aspergillus nidulans *[55]. Alternatively chitin deacetylases may also be involved in the digestion of chitin, whereby the resulting chitosan would be cleaved by chitosanases. It has been proposed that marine fungi use chitin deacetylases for the decomposition of chitin [53] according to the chitosan pathway [56].

Chitosanases (EC 3.2.1.132) have been described in families GH5, GH7, GH8, GH46, GH75, and GH80, the last three containing exclusively chitosanases. All presently characterized GH75 proteins are fungal chitosanases [57]. Members from this family are present in all filamentous ascomycete species, but absent in *R. oryzae*, in the yeast *S. cerevisiae *and in all described basidiomycete fungi (Additional File 2). In *R. oryzae*, one and two proteins from families GH8 and GH46 were identified, respectively (Additional File 2). Our analysis of the corresponding sequences from *R. oryzae *indicates that they are only distantly related to bacterial chitosanases (Additional File 1). Bacterial chitosanases are known to play a role in the degradation and utilization of exogenous chitosan, whereas fungal chitosanases are assumed to recycle chitosan from the FCW. Production of chitosanases has been demonstrated during autolysis in *Mucor rouxi *[58].

Fungal chitinases from family GH18 are essential for FCW remodelling during growth and development [59]. In *R. oryzae*, a total of 14 GH18 members was found, a value similar to what is found in known ascomycete and basidiomycete species (Additional File 2). All candidate proteins belonging to family GH18 are likely chitinases in *R. orzyae*, as previously suggested by phylogenetic analysis [18]. The *R. oryzae *genome contains four protein models belonging to family GH20 and comparison to functionally characterized enzymes revealed that these members are related to β-N-acetylglucosaminidases (EC 3.2.1.30) or β-N-acetylhexosaminidases (EC 3.2.1.52). Family CE9 which includes N-acetylglucosamine-6-phosphate deacetylases (EC 3.5.1.25) is important for the metabolism of chitin. *R. oryzae *has only one family CE9 member, a number similar to ascomycete and basidiomycete fungi. Fungal chitinases have been shown to have diverse roles, such as remodelling of their own cell wall and release of nutrients [60]. It remains unknown, whether these chitinolytic genes of *R. oryzae *also have a role in nutritional processes. *Rhizopus *and *Mucor *species have been shown to use chitinases for hyphal growth and autolysis [61,62]. For other zygomycete fungi such as *Mortierella *spp, chitinolytic activity has been linked to utilisation of nutrients [56].

#### α-1,3-Glucan degradation

Both α-1,3-glucosidase and glucan endo-α-1,3-glucosidase are involved in hydrolysis of α-1, 3-glucan. α-1,3-Glucosidases are classified in families GH31 and GH63 and glucan endo-α-1,3-glucosidase in families GH71 and 87. *R. oryzae *encodes five proteins likely to breakdown α-1,3-glucan degradation, a lower value compared to the filamentous ascomycetes and basidiomycetes, excepting *U. maydis*. Functional analysis suggests that three members of family GH31 and two of family GH63 are candidate α-glucosidases, but it is unknown if these α-glucosidases could deal specifically with α-1,3-glycoside linkages. Both families are known to contain different enzyme activities, such as α-glucosidases that hydrolyse α-1, 4-linkages. No proteins were found in families GH71 and GH87, known for bearing glucan endo-α-1,3-glucosidases, unlike several ascomycete and basidiomycete genomes that contain GH71 members.

#### β-1,3-Glucan and β-1,6-glucan degradation

β-1,3-Glucanases can be divided into exo-β-1,3-glucanases and endo-β-1,3-glucanases. Four of the seven GH5 members from *R. oryzae *were functionally annotated as a candidate β-glucosidase related to exo-1,3-β-glucanases (Additional File 1), while five of the six protein models of family GH3 were functionally annotated as a candidate β-glucosidase or exo-1,3-β-glucosidase. *R. oryzae *also encodes two proteins belonging to family GH72, a family typically containing GPI-anchored β-1,3-glucanosyltransferases. These enzymes are known to play a central role in the cross linking of cell wall β-1,3-glucans to other cell wall β-glucans [63]. The two *R. oryzae *GH72s are indeed related to characterized β-1,3-glucanosyltransglycosylases, and have a C-terminal GPI-anchor. The cell wall of zygomycetes consists of primarily of chitin and chitosan [52]. However, there is evidence that the cell wall of zygomycetes could contain small amounts of β-1,3-glucan. β-1,3-Glucans have been suggested to participate in the regulation of cell wall morphology in the zygomycete *M. rouxii *[64]. We have identified three putative 1,3-β-D-glucan synthases (family GT48) encoded by the *R. oryzae *genome. The likely presence of β-1,3-glucan might explain the recently detected susceptibility of *R. oryzae *to caspofungin, an antifungal agent which inhibits the 1,3-β-D-glucan synthase [65]. The presence of reduced amounts of β-1,3-glucans in the cell wall does not correlate with the significant numbers of 1,3-β-glucanases in the genome. This could indicate a possible role for the candidate β-1,3-glucanases in the degradation of cell walls of ascomycetes and basidiomycetes, whose cell walls are enriched in chitin and β-1,3-glucans [52,66,67].

β-1,6-Glucanases may be found in families GH5 and GH30. The *R. orzyae *genome contains seven protein models belonging to these two GH families (Table 3), but comparison to biochemically characterized enzymes allowed no clear identification of candidate β-1,6-glucanases in *R. orzyae *(Additional File 1). This agrees well with the lack of reports on the presence of β-1,6-glucan in the cell wall of *R. oryzae*.

### The hydrolytic potential of *R. oryzae *genome correlates with growth on fungal cell wall fractions and polysaccharides

**Figure 2:**
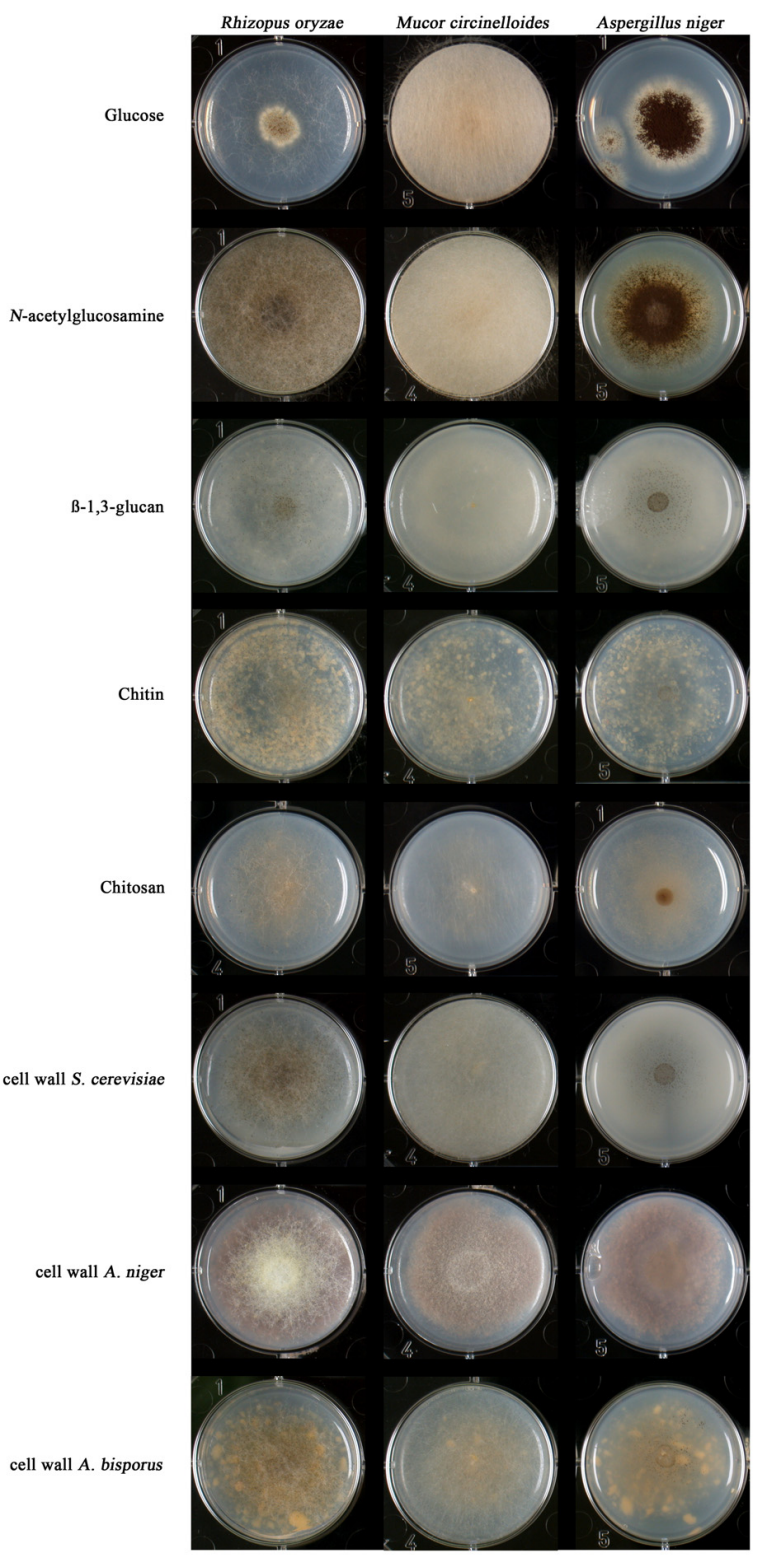
Growth of *R. oryzae*, *M. circinelloides *and *A. niger *on fungal cell wall fractions and polysaccharides.

The CAZy annotation results are consistent with growth data of *R. oryzae *on fungal cell wall polysaccharides and fractions thereof (Figure 2). Growth of *R. oryzae *on β-1,3-glucan, chitin and chitosan was comparable to the colony size and density on D-glucose. Significantly improved growth was observed on fungal cell wall fractions of *S. cerevisiae*, *A. niger *and *A. bisporus *(aggregated mycelium) (Figure 2), but no growth was observed on cell wall fractions of *R. oryzae *and *P. blakesleeanes *(data not shown). The zygomycete *M. circinelloides *showed a similar growth phenotype on cell wall polysaccharides and fractions. In contrast, poor growth of *A. niger *was observed on β-1,3-glucan, chitin, chitosan and cell wall fractions. All three species showed good growth on N-acetylglucosamine (Figure 2).

The chitinolytic and β-1,3-glucanolytic enzyme system of *R. oryzae *could have a role in either nutrition or fungal interactions. Fungal interactions may include inhibition of growth of coexisting fungi, a potential defence system for mycoparasitic competitors such as ascomycete *Trichoderma *spp. that are known to parasitize a range of fungi [68]. Degradation of chitin, chitosan and/or β-1,3 glucan in the environment can serves as a carbon and nitrogen source for fungi. Recycling of cell wall material of former fungal colonizers has been observed for primary and secondary basidiomycete species of wood during succession [69]. Growth of *R. oryzae *on chitin and chitosan suggest the ability to digest these polymers in the environment. Together with previous reported locations of isolation, e.g. the soil of salt-marshes [10], our observations are compatible with an adaptation of *R. orzyae *to inhabit coastal environments.

## Conclusions

The *R. oryzae *genome encodes a repertoire of CAZymes distinct of that of ascomycete and basidiomycete fungi, suggesting strong evolutionally differences and adaptation to the environment at the genomic level. In contrast to most ascomycetes and basidiomycetes, the set of CAZymes in the *R. oryzae *genome - supported by the growth profile - reveals the ability to use easily digestible sugars accompanied by an inability to degrade complex plant cell wall polysaccharides. The chitinolytic and glucanolytic enzyme system and the ability to degrade chitin, chitosan, β-1,3-glucan and ascomycete and basidiomycete cell walls suggest a role in both morphological processes and non-plant based nutrition. The differences in hydrolytic potential identified for *R. oryzae *suggest the development of unique strategies for polysaccharide degradation in the Zygomycota when compared to Basidiomycota and Ascomycota.

## Methods

### Strains and growth conditions

The fungal strains and culture conditions are listed in Suppl. Table 3. Carbon sources were added to the minimal medium (MM) containing 1,5% agar (Merck,101614) at the following concentrations: 1% (w/v) for soluble starch, guar gum, citrus pectin, inulin and beechwood xylan and 25 mM for D-glucose, D-mannose, D-galactose, D-galacturonic acid, D-fructose and D-xylose (Additional File 3). The pH of the medium was adjusted to 6.0. The plates were inoculated with 2 μl of 500 spores/μl or a mycelium plug (1 mm diameter). For growth comparison on fungal cell wall fractions and polysaccharides, of *Rhizopus oryzae*, *Mucor circinelloides *and *Aspergillus niger *were grown on plates containing *Aspergillus *minimal medium [70] and 1,5% agar with 25 mM glucose, or 0.5% (w/v) *N*-acetylglucosamine, chitin, chitosan, β-1,3-glucan (Additional File 3), or 0.5% (w/v) dried cell wall fractions of *Saccharomyces cerevisiae*, *Aspergillus niger*, *Agaricus bisporus*. Plates were inoculated with 5 μl of 500 *A. niger *spores/μl, or with a plug of *R. oryzae *or *M. circinelloides *mycelium. Cultures were grown for 3 days at 25°C.

### CAZy annotation

All 17,459 protein-encoding ORFs from the *R. oryzae RA 99-880 *genome were submitted to analysis using the CAZy annotation pipeline in a two-step procedure of identification and annotation. The identification step of CAZymes follows the procedures previously described [13] where sequences are subject to Blastp analysis [71] against a library composed of modules derived from CAZy, the positive hits are then subject to a modular annotation procedure that maps the individual modules against on the peptide using hits against libraries of catalytic and carbohydrate models derived from CAZy using BlastP or profile Hidden Markov models [71,72]. The results are augmented with signal peptide, transmembrane, and GPI predictions by human curators [73-75]. The fragmentary models and all models suspected of splicing prediction errors are identified. The functional annotation step involves BlastP comparisons against a library modules derived from biochemically characterized enzymes [13]. This manual comparison to identified functions yields three levels of detailed annotation: i) *candidate *activity, reflects a non-ambiguous assignment thanks to high levels of similarity and/or a large functional homogeneity of the hits; ii) *related to *activity, reflects a more distant similarity and/or a functional variability of the results; iii) *distantly related to *activity, reflects a weak degree of similarity to known activities. In most cases a generic activity (ex: α-glycosidase) was equally assigned.

### Fungal cell wall extractions

Cell wall fractions were isolated as described by Damveld *et al. *[76].

## Authors' contributions

All authors read and approved the final version of the paper. EB participated in the growth profiling and CAZy annotation and was the main author of the paper. IB contributed to interpretation of the results. JvdB and AW contributed to the experimental work. PMC and BH participated and supervised the CAZy annotation and contributed to the manuscript. RPdV supervised the experimental work and contributed to the manuscript.

## Supplementary Material

Additional file 1 Functional CAZy annotation. Click here for file

Additional file 2 Comparative analysis of the number of CAZy families related to plant polysaccharide degradation. Click here for file

Additional file 3 Strains culture conditions and carbon sources used. Click here for file
